# Roles of lysine-specific demethylase 1 (LSD1) in homeostasis and diseases

**DOI:** 10.1186/s12929-021-00737-3

**Published:** 2021-06-04

**Authors:** Dongha Kim, Keun Il Kim, Sung Hee Baek

**Affiliations:** 1 Department of Anatomy, College of Medicine, The Catholic University of Korea, Seoul, 06591 Republic of Korea; 2 Department of Biological Sciences, Sookmyung Women’s University, Seoul, 04310 Republic of Korea; 3 Creative Research Initiatives Center for Epigenetic Code and Diseases, Department of Biological Sciences, Seoul National University, Seoul, 08826 Republic of Korea

**Keywords:** Cancer, Hypoxia, Inflammation, Immunity, Neurodegenerative diseases

## Abstract

Lysine-specific demethylase 1 (LSD1) targets mono- or di-methylated histone H3K4 and H3K9 as well as non-histone substrates and functions in the regulation of gene expression as a transcriptional repressor or activator. This enzyme plays a pivotal role in various physiological processes, including development, differentiation, inflammation, thermogenesis, neuronal and cerebral physiology, and the maintenance of stemness in stem cells. LSD1 also participates in pathological processes, including cancer as the most representative disease. It promotes oncogenesis by facilitating the survival of cancer cells and by generating a pro-cancer microenvironment. In this review, we discuss the role of LSD1 in several aspects of cancer, such as hypoxia, epithelial-to-mesenchymal transition, stemness versus differentiation of cancer stem cells, as well as anti-tumor immunity. Additionally, the current understanding of the involvement of LSD1 in various other pathological processes is discussed.

## Introduction

Post-translational modifications of histone tails with various protein and non-protein tags are well-known mechanisms regulating chromatin structure and gene expression [1, 2]. These histone modifications are reversible and coordinated by the interplay of special “writer” and “eraser” enzymes. Lysine methylation of the histone tail was considered an irreversible process until lysine-specific demethylase 1 (LSD1, also known as KDM1A) was identified along with its histone demethylase activity against H3 lysine 4 (H3K4) [3]. LSD1 specifically demethylates mono- and di-methylated H3K4 through flavin adenine dinucleotide (FAD)-dependent amine oxidase activity, repressing gene expression by removing active histone marks (H3K4me2) from gene promoter regions [3] (Table 1). The repressive effect of LSD1 on nucleosomal substrates requires its association with other proteins, most notably the corepressor of REST (CoREST) protein [4, 5]. Structural studies revealed bivalent interactions between LSD1-CoREST and nucleosomes, during which LSD1 recognizes the H3 tail, while CoREST interacts with DNA [6]. In addition to CoREST, LSD1 was found in protein complexes with CtBP [7] and NuRD [8]. Further, various transcription factors have been reported to recruit LSD1 to gene promoters as well as enhancers, leading to H3K4 demethylation [9].

**Table 1:** Targets of LSD1 and their effect on gene expression

Targets	Residue	Functions	Effect	References
Histone H3	K4me2	Repression with CoREST, CtBP and NuRD	Gene repression	[6–8]
Histone H3	K9me2	Derepression of AR target genes with FOXA1	Gene activation	[10, 11]
Histone H4	K20me1	Regulation of memory formation (LSD1n*)	Gene activation	[19]
DNMT1	K1096	Maintenance of global DNA methylation through stabilization of DNMT1	Gene activation	[8]
FOXA1	K270	Derepression of AR target genes through stabilization of FOXA1	Gene activation	[11]
HIF-1α	K32	Regulation of tumor angiogenesis through stabilization of HIF-1α	Gene activation	[14]
p65	K314/315	Regulation of inflammatory response through stabilization of p65	Gene activation	[15]
AGO2	K726	Inhibition of anti-tumor immunity through stabilization of AGO2	Gene repression	[16]
SOX2	K42/117	Maintenance of pluripotency through stabilization of SOX2	Gene activation	[17]

^*^ neuron-specific isoform of LSD1

Listed are the target substrates of LSD1 and the cellular functions and effects of gene expression

In contrast to its repressive role at H3K4, LSD1 functions as a transcriptional coactivator when bound to the androgen receptor (AR) via demethylation of repressive histone marks at H3K9, resulting in the derepression of AR target genes [10]. In addition, a recent study revealed the LSD1-mediated demethylation of FOXA1, a crucial cofactor for AR chromatin accession. LSD1 stabilized FOXA1, resulting in the expression of AR target genes and tumor growth [11]. Thus, LSD1 appears to increase AR-mediated transcription by regulating chromatin status through the demethylation of H3K9 as well as non-histone cofactors. As in the case of FOXA1, LSD1 demethylation targets include non-histone transcriptional regulatory proteins, such as transcription factors and cofactors. The outcomes of non-histone protein methylation vary depending on the target and context, but one important consequence is the regulation of protein stability [12, 13]. Many studies have reported that SET7/9-mediated monomethylation of non-histone proteins, including DNMT1, HIF-1α, p65, AGO2, SOX2, and FOXA1, induces their ubiquitin-dependent proteasomal degradation, while LSD1, by reversing this modification, stabilizes substrate proteins against degradation [8, 11, 14–17]. Lastly, neuron-specific alternative splicing forms of LSD1 have been identified with distinct target specificity from that of the original form [18, 19]. The neuronal isoform of LSD1 demethylates H3K9me2 in collaboration with supervillain, does not exhibit H3K4me2 demethylation activity, and is involved in neuronal differentiation [18]. In addition, this isoform exhibits altered intrinsic substrate specificity against H4K20 methylation, a newly identified histone substrate, and promotes the transcription of genes involved in learning and memory [19].

LSD1 plays pivotal roles in various physiological and pathological processes via its demethylase activity against both histone and non-histone targets. In this review, we discuss the current understanding of LSD1’s involvement in different pathological processes. We focus on the molecular mechanisms through which LSD1 contributes to the maintenance of homeostasis and disease progression.

## LSD1 in cancer

LSD1 is involved in various stages of cancer, including development, progression, metastasis, and recurrence after therapy. Although overexpression of LSD1 has been reported in various cancer types and correlated with poor overall survival in patients [20–24], *LSD1* does not appear to be a potent oncogene. Instead, LSD1 supports cancer progression by regulating gene expression in cancer cells in favor of adaptation to the tumor microenvironment. Further, LSD1 is considered a drug target in cancer, and numerous LSD1 inhibitors have been developed, some of which are currently undergoing clinical trials for the treatment of hematological cancers as well as lung cancer and other solid tumors [25]. LSD1 inhibitors are not within the scope of this work and have been thoroughly discussed in other reviews [25, 26]. We focus on several aspects of cancer wherein LSD1 plays a disease-promoting role, such as hypoxia, the epithelial-to-mesenchymal transition (EMT), cancer stemness and differentiation, as well as antitumor immunity.

### Regulation of hypoxia by LSD1

Eukaryotic cells including cancer cells have an elaborate system for adaptation to low oxygen levels (hypoxia) in their microenvironment [27]. Hypoxia induces the transcriptional activation of various genes via the stabilization of hypoxia-inducible factor (HIF)-1α [27, 28]. In the normal physiological oxygen state (normoxia), a series of enzymatic reactions initiated by oxygen-sensing prolyl-hydroxylases (PHDs) maintain HIF-1α at low levels. PHD enzymes hydroxylate HIF-1α at its proline residues, which serves as a signal for its ubiquitylation by the von Hippel-Lindau (VHL)-containing E3 ubiquitin ligase complex [29] and subsequent proteasomal degradation. Under hypoxic conditions, the lack of oxygen inhibits PHD function, leading to HIF-1α stabilization, nuclear localization, and transcriptional activation. In addition to the oxygen-dependent regulation of HIF-1α stability, oxygen-independent mechanisms, such as the CHIP-HSP70 or RACK1-HSP90 pathways, are known to degrade HIF-1α [30, 31]. Furthermore, various post-translational modifications, including acetylation, methylation, and SUMOylation, are critical for the regulation of HIF-1α stability and function [32, 33].

**Fig. 1:**
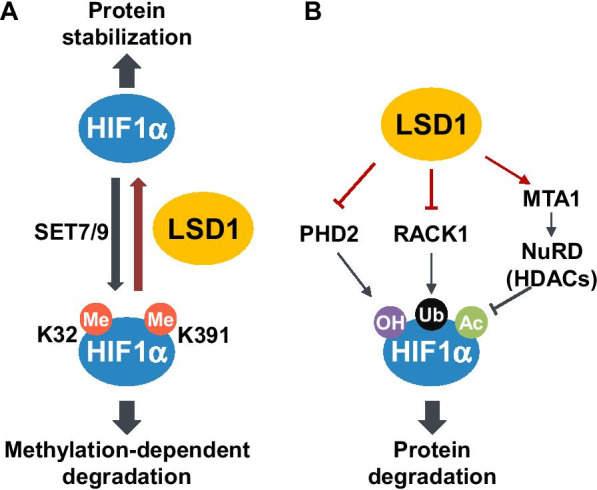
Regulation of the stability of HIF-1α by LSD1 in hypoxia. **A** Methylation of HIF-1α Lys32 or Lys391 residue by SET7/9 induces ubiquitination and degradation of HIF-1α. LSD1 removes the corresponding methyl groups and thereby stabilizes HIF-1α. **B** LSD1 prevents PHD2-induced hydroxylation and enhances K532 deacetylation of HIF-1α, thereby stabilizing HIF-1α. In addition, demethylation of RACK1 by LSD1 inhibits the interaction of RACK1 with HIF-1α, consequently stabilizing HIF-1α

The role of LSD1 as a positive factor in the hypoxic regulation of HIF-1α stability and transcriptional activity via demethylation of HIF-1α and HIF-1α-interacting protein RACK1 has been well studied [14, 34–36]. Monomethylation of the HIF-1α Lys32 residue by SET7/9 mediates HIF-1α ubiquitylation and degradation (Fig. 1A) [14, 34]. As the E3 ubiquitin ligase that is targeting monomethylated HIF-1α is not yet known, its identification adds to our understanding of HIF-1α stability regulation. Further, this E3 ubiquitin ligase may be a promising drug target for HIF-1α suppression via PROteolysis Targeting Chimera (PROTAC) technology [37, 38], especially in VHL-defective cancers. Under hypoxic conditions, LSD1 maintains HIF-1α stability and hypoxia-responsive gene expression by counteracting SET7/9-mediated HIF-1α monomethylation [14]. A mouse model harboring a lysine-to-alanine substitution at HIF-1α Lys32, which prevents its monomethylation, exhibited much higher HIF-1α levels, upregulated hypoxia-inducible gene expression, as well as enhanced tumor growth and angiogenesis [14], suggesting that monomethylation at this lysine residue is a critical regulator of HIF-1α function. Lysine monomethylation of HIF-1α also occurs at position 391 and is regulated by the interplay between SET7/9 and LSD1 (Fig. 1A) [35]. This site is in close proximity to the oxygen-dependent degradation domain (ODDD) of HIF-1α, and methylation by SET7/9 enhances VHL-mediated HIF-1α ubiquitylation [35]. LSD1 increases HIF-1α stability by inhibiting the methylation. In addition, LSD1 prevents PHD2-induced hydroxylation and enhances K532 deacetylation (Fig. 1B) [35]. LSD1 increases MTA1 (Metastasis Associated 1, a component of NuRD complex) level as well as HDAC activity in NuRD complex, resulting in the decrease of Arrest defect 1 (ARD1)-mediated K532 acetylation. These events stabilize HIF-1α. Another level of LSD1-mediated HIF-1α stability regulation is through an oxygen-independent mechanism via demethylation of RACK1 (Fig. 1B) [36]. RACK1 was identified as a HIF-1α-interacting protein and was shown to recruit an E3 ubiquitin ligase independently of oxygen status [30]. LSD1 demethylates the Lys271 residue of RACK1, inhibiting its interaction with HIF-1α and thus stabilizing the latter [36]. Low cofactor FAD levels attenuate LSD1 demethylase activity during prolonged hypoxia and suppress HIF-1α through RACK1-mediated degradation [36]. In summary, LSD1 increases HIF-1α stability under hypoxic conditions via three mechanisms. First, direct demethylation of HIF-1α stabilizes it against methylation-mediated degradation. Second, LSD1 indirectly inhibits HIF-1α hydroxylation-mediated degradation. Third, LSD1 demethylates RACK1 and inhibits the RACK1-mediated degradation of HIF-1α. Thus, LSD1 inhibition is a promising strategy for preventing cancer cell adaptation to the hypoxic microenvironment.

While most studies on the function of LSD1 in hypoxia have focused on its regulation of HIF-1α stability, Sakamoto et al*.* suggested that histone demethylation by LSD1 is partially involved in cancer cell metabolic reprogramming [39]. They observed a shift in the metabolic balance from glycolytic to mitochondrial respiration upon LSD1 depletion in hepatocellular carcinoma cells. Reduced glucose uptake and glycolytic activity were correlated with the decrease of HIF-1α levels, while elevated mitochondrial respiration in parallel to the increase of methylated H3K4 in the promoter region of respiratory genes. In various independent studies, LSD1 was shown to play a role in the cellular response to hypoxia [14, 35, 36, 39, 40]. Since LSD1 levels and activity were associated with angiogenesis, tumor growth [35], as well as the metabolic shift toward glycolysis [39], LSD1 inhibitors are promising candidates for suppressing cancer progression in relation to the hypoxic response.

### LSD1 in the epithelial-to-mesenchymal transition (EMT)

The EMT is an essential process allowing solid cancer cells to gain migratory potential and relocate from their original location [41, 42]. The process involves repression of epithelial marker genes, such as *E-cadherin*, and the activation of mesenchymal marker genes, including *vimentin*. Various transcription factors involved in the EMT have been characterized, including the SNAIL, TWIST, and ZEB families [41, 42]. Among them, SNAIL is known to recruit a number of epigenetic regulators in order to establish the heterochromatin state in the promoter region of epithelial marker genes [43]. Further, LSD1 plays a critical role in the initiation of this epigenetic repressive state.

LSD1 was initially proposed as a negative regulator of EMT and invasion in breast cancer cells, acting through the inhibition of TGF-β signaling gene expression via a complex formed with NuRD [8]. However, many other reports later suggested a role of LSD1 in promoting EMT across various types of cancer. The EMT-promoting role of LSD1 was first described when it was identified as a SNAIL-binding protein [43, 44]. SNAIL interacts with LSD1 through the N-terminal SNAG domain and recruits LSD1 to target gene promoters (e.g., SNAIL gene promoter) via binding to E-box consensus sequences, where LSD1 strips H3K4me2 histone marks [43, 44]. LSD1 simultaneously recruits the CoREST repressor complex to the promoter for transcriptional repression [43]. Further, LSD1 contributes to the enhanced stability of the SNAIL protein preventing its GSK3β-mediated degradation via the ubiquitin–proteasome system [45]. Without LSD1 binding, SNAIL failed to repress target gene expression [43], highlighting the critical role of the former in SNAIL-mediated epithelial gene repression. SNAIL family member SLUG (also called SNAIL2) also interacts with LSD1 via its SNAG domain [46]. LSD1 together with SNAIL/SLUG were involved in the repression of cancer-related genes other than epithelial-specific genes depending on the cancer type. Examples included BRCA1 (Breast cancer 1; the tumor suppressor) in triple-negative breast cancer cells [46] and NDRG1 (N-myc downstream-regulated gene 1; the metastasis suppressor) in neuroblastoma with the *MYCN* oncogene amplification [47]. The contribution of SNAIL-LSD1 to the development of acute myeloid leukemia (AML), a hematological malignancy, suggests that the complex is also involved in aspects of cancer progression other than the EMT [48].

Genome-wide analysis of epigenomic reprogramming during the EMT confirmed the pivotal role of LSD1. TGF-β-induced EMT in mouse hepatocytes was accompanied by a reduction in heterochromatin mark H3K9me2 levels in parallel to an elevation in the euchromatin marks H3K36me3 and H3K4me3 [49]. Importantly, the major contributor to this reprogramming was found to be LSD1. In addition, the increased motility and chemoresistance of TGFβ-treated cells were LSD1-dependent [49]. Thus, LSD1 acts a critical epigenetic EMT regulator via both the repression of epithelial gene expression and the activation of mesenchymal gene expression. Genome-wide analysis revealed a general shift of chromatin structure toward a more open state with increased euchromatin histone marks, which was not in agreement with the repression of epithelial genes during the EMT. One possible scenario is that the repression of epithelial-specific expression occurred because of SNAIL/LSD1-induced local repressive chromatin states rather than a broader genomic state of repression.

Post-translational modification of LSD1 contributes to its activation during the EMT. Phosphorylation at the Ser112 residue is critical for EMT activation [50, 51]. In nude mice with LSD1 mutant-expressing MDA-MB-231 tumors, ectopic overexpression of wild-type LSD1 or a phosphorylation-mimicking LSD1-S111D (originally reported as S112 referring to mouse LSD1, while S111 is the human LSD1 residue) enhanced metastasis, whereas overexpression of a phosphorylation-defective S112A mutant did not change the metastatic potential of cancer cells [50]. The kinase responsible for phosphorylation at the site was suggested to be chromatin-anchored PKC-θ [51], although LSD1-S112 was originally identified as a target for PKCα in relation to circadian regulation [52] and inflammation [15]. LSD1, together with PKC-θ, localized to mesenchymal gene promoters and enhanced gene expression, while LSD1 inhibition repressed mesenchymal gene induction [51]. In contrast to the phosphorylation-mediated activation of LSD1 in EMT, acetylation was shown to negatively regulate EMT-promoting function of LSD1 in epithelial cells, thereby attenuating the EMT [53]. Acetyltransferase MOF, which is highly expressed in epithelial cells, acetylates LSD1 at multiple lysine residues, interfering with its association with chromatin and thus compromising the EMT-promoting function of LSD1 [53].

Since LSD1 was identified as a critical player in the EMT, various approaches have been employed for blocking the EMT via inhibition of either LSD1 activity or the SNAIL interaction [54, 55]. Structurally, the N-terminal SNAG domain of SNAIL mimics histone H3, allowing for its interaction with LSD1 [56]. Thus, a SNAG mimicry peptide blocked the SNAIL-LSD1 interaction [55]. In addition, LSD1 demethylase inhibitor tranylcypromine (Parnate) also suppressed their interaction [54, 55]. Further, the treatment of various cancer cell lines with these two inhibitory molecules resulted in an increased expression of E-cadherin and the suppression of motility and invasiveness [54, 55]. LSD1 inhibitors Pargyline and GSK-LSD1 were applied to restrict the EMT in prostate cancer cells and oral squamous cell carcinoma, respectively. These inhibitors activated epithelial genes and repressed mesenchymal gene expression, in turn suppressing or delaying progression [19, 57]. In summary, a considerable amount of evidence has confirmed the LSD1-mediated promotion of the EMT in various types of cancer. Accordingly, LSD1 inhibitors have been successfully applied to suppress the EMT process and cancer progression in these preclinical studies.

### Maintenance of cancer stemness and regulation of differentiation by LSD1

The critical role of LSD1 in the regulation of stemness and differentiation was first reported in embryonic stem cells (ESCs) of humans and mice [58, 59]. LSD1 repressed the expression of several critical developmental genes by regulating the methylation status of H3K4 via its enzymatic activity [58]. As a result, LSD1 knockdown induced the differentiation of human ESCs via the early expression of mesodermal and endodermal marker genes, in parallel to increased levels of H3K4 di- and tri-methylation [58]. LSD1 depletion in mouse ESCs leads to their incomplete differentiation due to the partial silencing of ESC genes, as LSD1 was crucial for the decommissioning of ESC-specific enhancers during differentiation [59]. Many LSD1-regulated genes are its indirect targets, wherein LSD1 recruitment is mediated by master ESC transcription factors, including OCT4, SOX2, and NANOG [58, 59]. In addition to its role in ESCs, LSD1 is involved in the differentiation of adult stem cells in various tissues, including myogenic differentiation [60], adipogenesis [61], hematopoiesis [62, 63], and epithelial differentiation [64]. Taken together, LSD1 is a critical epigenetic factor that maintains the self-renewal potential of stem cells and regulates their cellular differentiation.

In tumors, a small population of cells containing stem cell-like properties (i.e., self-renewal, long-term growth, and drug resistance) have been identified and termed cancer stem cells (CSCs) [65, 66]. After the characterization of LSD1 as a pivotal regulator contributing to embryonic stem cell stemness and differentiation, various groups have explored its role in CSCs. LSD1 inhibition via small-molecule compounds selectively suppressed the growth of stem-like cancer cells in teratocarcinoma, embryonic carcinoma, and testicular seminoma, without significant growth inhibition observed in non-pluripotent cancer cells [67]. Since this initial observation, various studies have elucidated the function of LSD1 and the consequences of LSD1 deletion or pharmacological inhibition in CSCs from a variety of cancer types. Although the mechanism of LSD1 function in CSCs varies and has not been clearly established, it is related to the regulation of stemness and differentiation, similarly to its role in ESCs. Thus, LSD1 inhibition is to be accompanied by reduced stemness, cellular differentiation, and/or diminished drug resistance in CSCs. Pharmacological LSD1 inhibition in small cell lung carcinoma (SCLC), a notoriously drug-resistant lung cancer type, had cytostatic effects with a delayed onset of growth both in vitro and in xenograft models [68]. LSD1 caused a change in the cellular state (i.e., inducing differentiation) via neuroendocrine marker gene expression changes, which are a molecular feature of SCLCs [68]. Unfortunately, not all SCLC cell lines respond to LSD1 inhibition, and the exact mechanism through which LSD1 contributes to the CSC phenotype has not been elucidated. In squamous cell carcinoma (SCC), LSD1 function was associated with the stem cell factor SOX2. LSD1 inhibitors selectively suppressed the growth and promoted the differentiation of SOX2-positive, but not SOX2-negative, SCCs, in conjunction with the upregulation of differentiation-associated genes [69].

In leukemia, LSD1 inhibition promotes cell differentiation. In MLL-AF9-driven leukemia, LSD1 sustained the expression of oncogenic genes maintaining stem cell potential in concert with the MLL-AF9 oncoprotein. Further, knockdown or pharmacological inhibition of LSD1 attenuated leukemia stem cell potential and induced differentiation [70]. Moreover, inhibition successfully induced cell fate transition in other types of leukemia. In particular, inhibiting LSD1 triggered the differentiation of non-acute promyelocytic leukemia (APL)-type AML cells, which do not respond to all-*trans*-retinoic acid (ATRA)-mediated differentiation, into ATRA-sensitive cells [71]. Interestingly, recent evidence revealed that even though LSD1 inhibition was not effective in APL cell treatment, it sensitized APL cells to physiological doses of retinoic acid so that combination treatment of LSD1 inhibitor and retinoic acid extended the survival of leukemic mice [72]. However, in this case, the demethylase activity of LSD1 was not correlated with its retinoic acid-mediated sensitizing ability, and LSD1 inhibition disrupted the interaction between LSD1 and GFI1 [72]. Pharmacological dissociation of LSD1 from the LSD1-GFI1 complex has been reported as crucial for the differentiation of AML cells [73, 74]. In addition, pharmacological inhibition of LSD1 in Merkel cell carcinoma, a primary neuroendocrine carcinoma of the skin, induced cell cycle arrest and cell fate change to the normal Merkel cell phenotype accompanied by the de-repression and activation of the neuronal transcription program [75]. Thus, LSD1 is a critical player in the maintenance of stemness, and LSD1 inhibition has been demonstrated as efficient for the treatment of differentiation-prone cancer types in pre-clinical models.

LSD1 is likely to regulate the methylation status of lineage- or cancer type-specific gene sets rather than the global chromatin methylation status in CSCs [64, 70, 71, 76]. The question is how LSD1 selectively targets gene-specific promoters. One plausible explanation is that LSD1 works together with or regulates stem cell factors in a subset of CSC types. One candidate factor, SOX2, was reported to cooperate with LSD1 in lung cancer [69], HER2-positive breast cancer [77], human ovarian teratocarcinoma [17], and pluripotent cancer cells, including teratocarcinoma, embryonic carcinoma, and seminoma CSCs [67]. LSD1 inhibition repressed the expression of *SOX2* in lung squamous cell carcinomas, resulting in the suppression of SOX2-mediated oncogenic potential [69]. In human ovarian teratocarcinomas, SOX2 was susceptible to monomethylation-mediated degradation, which was reversed by LSD1, and thus LSD1 inhibition caused SOX2 destabilization [17]. LSD1 inhibition selectively blocked CSC-driven mammosphere formation in SOX2-driven CSCs [77]. Another stem cell factor, OCT4, was also suggested as a stemness-regulating factor working in concert with LSD1. The LSD1-OCT4 interaction in CSCs was suggested to maintain enhancers susceptible to reactivation, leading to the abnormal expression of pluripotency-related genes [78]. In LGR5-positive hepatocellular carcinoma, LGR5 and LSD1 appeared to enhance each other's expression, maintaining the stem cell characteristics of carcinoma cells [79]. A key feature of neuroblastoma is the impaired neuronal differentiation in parallel to the high expression of myelin transcription factor 1 (MYT1). Knockdown of MYT1 induced neuro-differentiation in neuroblastoma cells [80]. Pharmacological inhibition of MYT1 interaction partner LSD1 had similar effects. Therefore, the roles of LSD1 in CSCs need to be considered together with those of its interaction partners, which are most likely lineage- or cancer type-specific transcriptional regulators.

### LSD1 in anti-tumor immunity

The immune surveillance system of the human body continuously identifies and clears tumor cells through a multi-step process. In brief, these steps include the recognition of tumor-associated antigens by dendritic cells (DCs), antigen presentation via major histocompatibility complexes on the DC surface, the migration of DCs to lymphoid organs, DC-T cell contact resulting in T cell activation, relocation of activated T cells to peripheral tumor sites, and finally, the recognition and cytotoxic T cell-mediated destruction of cancer cells [81]. In addition, tumor antigen-specific B cells proliferate, differentiate, and produce antibodies to help with the destruction of cancer cells [81]. However, cancer cells employ various mechanisms to perturb and escape from immune surveillance, including immune editing [82]. Therefore, various approaches for overcoming the immune escape of cancer cells are being actively investigated.

**Fig. 2:**
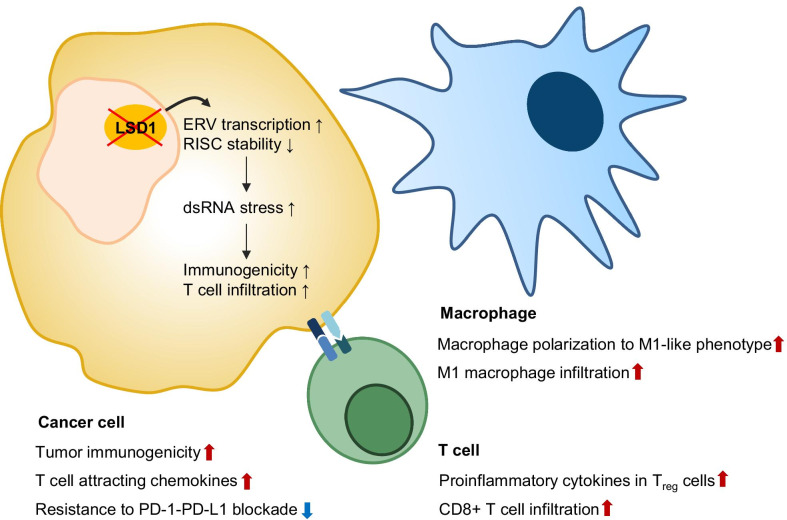
The effect of LSD1 inhibition on anti-tumor immunity. LSD1 inhibition in cancer cells enhances tumor immunogenicity and secretion of chemokines that attract T cells, while reversing the resistance to PD-(L)1 blockade. LSD1 inhibition upregulates pro-inflammatory cytokines in Treg cells, promotes the polarization of macrophages into the anti-tumor M1-like macrophages, and enhances the infiltration of these macrophages and CD8 + T cells into the tumor

Tumor immunotherapy is a major antitumor treatment approach that has recently emerged into the clinical spotlight. It was not long ago that LSD1 inhibitors were also shown to be effective in antitumor immunotherapy (Fig. 2). Based on the fact that targeting epigenetic regulators can boost the antitumor immune response, researchers screened compounds targeting chromatin factors that upregulate the expression of endogenous retroviral element (ERV)—as well as type I interferon (IFN)-responsive genes, and identified GSK-LSD1 as a hit [16]. Further analysis revealed that LSD1 inhibition caused ERV expression in parallel to H3K4me2 upregulation at various ERV regions, resulting in the downregulation of RNA-induced silencing complex (RISC), which otherwise clears double-stranded ERV transcripts. These two phenomena in turn enhanced the cellular response to dsRNA by activating type I IFN signaling. In addition, LSD1 depletion or inhibition enhanced tumor immunogenicity, resulting in increased T cell infiltration of tumors. Furthermore, LSD1 inhibition sensitized PD-(L)1-resistant tumor cells to checkpoint blockade [16]. This was the first report to highlight the potential application of LSD1 inhibition in cancer immunotherapy, especially for the treatment of tumors that are poorly immunogenic and resistant to PD-(L)1 blockade. In agreement with this observation, various researchers reported the pro-immunogenic effects of LSD1 inhibition in tumors, which lead enhanced immune checkpoint blockade efficacy. LSD1 inhibition in triple-negative breast cancer (TNBC) increased the expression of CD8+ T cell-attracting chemokines and PD-L1 in parallel to upregulated H3K4me2 marks at the respective gene promoter regions, resulting in enhanced CD8+ T cell infiltration at tumor sites [83]. Further, combination treatment with LSD1 inhibitor and an anti-PD-1 antibody in TNBC xenograft mice amplified PD-1 blockade efficacy [83]. Enhanced antitumor immune responses, including T cell infiltration, after LSD1 inhibition have also been reported in SWI/SNF-mutated ovarian cancers as well as in a 4T1 melanoma mouse model [84, 85]. Interestingly, LSD1 deficiency of CD8+ T cells in a murine melanoma model resulted in lower tumor growth, higher PD-1 levels in the tumor-infiltrating population, and no significant difference in overall health compared to wild-type mice [86]. CoREST complex inhibitor corin, which is derived from a class I HDAC inhibitor and an LSD1 inhibitor [87], induced the expression of proinflammatory cytokine genes in Tregs, resulting in increased CD8+ T cell tumor infiltration and reduced tumor burden [88]. Therefore, LSD1 inhibition seems to exert anti-tumor effects on both tumor and immune cells. Further, it is of interest to observe whether a single LSD1 inhibitor can exert similar effects on Treg cells as the dual CoREST inhibitor corin.

In addition to modulating tumor immunogenicity and PD-(L)1 blockade, LSD1 inhibition potentiates antitumor immunity by activating innate immune cells, including macrophages and natural killer cells. In a xenograft model of MDA-MB-231 breast cancer in BALB/c nude mice, LSD1 inhibition via phenelzine resulted in increased infiltration of anti-tumor M1 macrophages in the tumor microenvironment [51]. Further analysis of the effect of LSD1 inhibitors on macrophage polarization programs revealed that phenelzine could target both FAD and CoREST binding domain, while catalytic inhibitor GSK2879552 could not switch the macrophage polarization program toward an anti-tumoral M1-like phenotype [89]. In contrast, catalytic inhibitor GSK-LSD1, but not scaffold inhibitors, increased natural killer cell-mediated tumor regression in a mouse model of pediatric high-grade glioma [90]. One concern was the cytostatic and cytotoxic effects of scaffold-type LSD1 inhibitors on natural killer cells, which did not affect T cells [90]. This was due to the impaired metabolism and glutathione depletion in inhibitor-treated natural killer cells, as glutathione supplementation rescued their cytolytic function [91].

Taken together, studies have thoroughly demonstrated the beneficial effects of LSD1 inhibition on antitumor immunotherapy. In cancer, LSD1 inhibition enhances tumor immunogenicity and the secretion of chemokines, while reversing resistance to PD-(L)1 blockade. Further, LSD1 inhibition increases proinflammatory cytokine expression in Treg cells and enhances tumor CD8+ T cell infiltration. Antitumor M1-like macrophage polarization and infiltration were also upregulated by LSD1 inhibition. As LSD1 inhibitors have therapeutic potential within immunotherapy, further in-depth research is crucial for determining which type of LSD1 inhibitors (catalytic inhibitor vs. scaffolding inhibitor) is to be used and what type of immunotherapy it should be combined with.

## Roles of LSD1 in inflammatory diseases

**Fig. 3:**
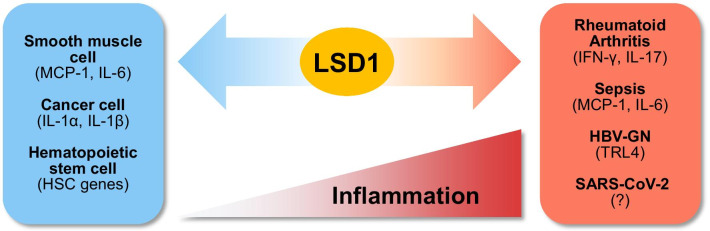
LSD1 functions as a key epigenetic regulator in inflammatory disease. LSD1 controls gene expression in two ways during inflammation. LSD1 increases the expression levels of inflammatory response genes by acting as a positive regulator in inflammatory diseases such as rheumatoid arthritis (RA), sepsis, hepatitis B virus-associated glomerulonephritis (HBV-GN), and SARS-CoV-2 infection. By contrast, LSD1 functions as a negative regulator of the inflammatory response that decreases the expression of cytokine genes in smooth muscle cells (SMCs), cancer cells, and hematopoietic stem cells (HSCs)

The inflammatory response is the primary defense mechanism mediated by the immune system. However, in order for the inflammatory response to function efficiently, its induction and termination must be tightly regulated, as excessive and prolonged inflammation leads to the development of chronic inflammatory diseases [10, 92, 93]. It has been reported that the epigenetic regulation of gene expression during inflammation is crucial in inflammatory disease pathogenesis [94–97]. LSD1 functions as an epigenetic regulator of the inflammatory response (Fig. 3) [98–100]. It regulates the expression of pro-inflammatory genes through different mechanisms depending on the cell type and immune signal. LSD1 repressed pro-inflammatory cytokines *MCP-1* and *IL-6* induced by diabetes in smooth muscle cells (SMCs) through the regulation of H3K4me2 [99]. Further, LSD1 was downregulated in SMCs isolated from a diabetic mouse model (*db*/*db*), resulting in the increased H3K4 methylation of NF-κB response elements near the *MCP-1* and *IL-6* promoters [99]. In cancer cell lines MDA-MB231 and HepG2, LSD1 repressed pro-inflammatory cytokine genes *IL-1α*, *IL-1β*, and *IL-6* [98]. LSD1 knockdown upregulated H3K4me2 at the promoters of *IL-1α*, *IL-1β*, and *IL-6*, while H3K9me2 levels decreased. LSD1 functioned in synergy with HDAC1 to control H3K9/14 acetylation [98]. In addition, LSD1 was reported to act as a negative epigenetic regulator of inflammatory responses in hematopoietic stem cells (HSCs) during endotoxin shock. *LSD1*^*fl/fl*^:*Mx-Cre* mice (*LSD1*^*−/−*^), which were LSD1-depleted via Poly(I:C), exhibited a dysregulated inflammatory response, leading to hyperinflammation and sudden death because of a cytokine storm. When LSD1 was restored in HSCs, these were resistant to LPS-induced septic shock. LSD1 directly modulated HSC gene expression through H3K4me2 regulation, thus preventing excessive inflammatory reactions [100].

LSD1 functions not only as a negative regulator of the inflammatory response but may also promote it [15, 101, 102]. In rheumatoid arthritis (RA), one of the most frequently occurring chronic inflammatory diseases, LSD1 acts as a positive regulator of CD4+ T cell activation [101]. Upregulated LSD1 expression was observed in CD4+ T cells within the synovial fluid (SF) of patients with active RA. Further, the production of both IFN-γ and IL-17 was significantly decreased by LSD1 knockdown in these cells. In the collagen-induced RA mouse model, LSD1 knockdown prevented RA development [101]. LSD1 was phosphorylated by PKCα in response to LPS stimulation of bone marrow-derived macrophages (BMDMs). LSD1 phosphorylation-defective mice (*Lsd1*^*SA/SA*^) were highly resistant to LPS-induced inflammation, acute lung injury, and mortality [15]. Further, LPS-induced LSD1 phosphorylation was required for the prolonged activation of NF-κB target genes. Interestingly, the NF-κB target gene activation associated with LPS-induced LSD1 phosphorylation was independent of its demethylase activity against H3K4me2 and H3K9me2 [15]. LPS-induced LSD1 phosphorylation was responsible for both p65 demethylation and binding, enhancing p65 protein stability. Inhibition of PKCα or LSD1 activity in mice attenuated sepsis-induced mortality. Therefore, phosphorylation by PKCα is critical for LSD1-mediated epigenetic regulation of the inflammatory response [15]. LSD1 is highly expressed in the renal tissue of hepatitis B virus-associated glomerulonephritis (HBV-GN) patients. Further, LSD1 promoted HBV-induced pro-inflammatory cytokine release in HK-2 cells, a human renal tubular epithelial cell line. LSD1 regulated expression by removing methylation from H3K9 at the promoter of inflammatory response-related gene *Tlr4*. Tranylcypromine, an LSD1 inhibitor, inhibited the TLR4-NF-κB-JNK signaling axis and decreased renal inflammation in HBV-infected transgenic mice. Taken together, LSD1 functions as a novel positive regulator of renal inflammation [102].

SARS-CoV-2, responsible for the current COVID-19 pandemic, is transmitted to the respiratory tract, causing severe respiratory syndrome, among which pneumonia is the most frequent. SARS-CoV-2-induced pneumonia causes a cytokine storm to develop rapidly, leading to death. Therefore, the epigenetic suppression of excessive inflammation represents a new avenue for the development of relevant therapeutics. Epigenetic regulators, such as LSD1, may thus represent promising therapeutic targets for the treatment and prevention SARS-CoV-2-associated pneumonia.

## Roles of LSD1 in thermogenesis and adipogenesis

Metabolic conditions, such as obesity and diabetes, are associated with the dysregulation of metabolism-associated gene expression [103, 104]. The best studied function of LSD1 in energy metabolism is its role in the positive regulation of thermogenic potential in adipocytes. LSD1 expression was decreased in the white adipose tissue (WAT) from *ob/ob* mice. In normal mice, its expression was induced in WAT after cold exposure or β-adrenergic stimulation [105]. Further, LSD1 upregulation enhances mitochondrial function, inducing the expression of genes related to oxidative phosphorylation (OXPHOS) in WAT via nuclear respiratory factor 1 (Nrf1) [105]. Transgenic mice with increased LSD1 expression exhibited partial browning of the WAT with reduced weight gain in high-fat diet-fed conditions [105]. In addition to the WAT, LSD1 contributes to the development, maintenance, and state conversion (active thermogenic vs. dormant) of the brown adipose tissue (BAT) by controlling the expression of metabolic target genes [106–108]. BAT-specific LSD1 conditional deletion resulted in the morphological whitening of the BAT, reduced thermogenic potential, and increased obesity [106–108]. With respect to the mechanism, LSD1 has a dual regulatory role of gene expression in the BAT, with upregulation of BAT-specific genes and downregulation of WAT-selective genes. The upregulation of BAT-specific genes is mediated by the interaction of LSD1 with transcription factors such as Zfp516, a BAT-enriched and cold-inducible transcription factor, and Nrf1 [106, 107]. Zfp516 recruits LSD1 to the promoter region of BAT-specific genes such as *UCP1*, and LSD1 demethylates H3K9 leading to transcriptional activation [107]. The Nrf1–LSD1 complex also targets H3K9 marks and induces BAT-specific gene transcription [106]. By contrast, repression of WAT-selective genes is achieved by the interaction of LSD1 with PRDM16, a key transcriptional coregulator in adipocytes, and through the LSD1–CoREST complex [106, 108]. Although LSD1–PRDM16-mediated repression of WAT-selective genes correlates with H3K4 demethylation [108], the LSD1–CoREST complex has a dual regulation function on both H3K4 and K3K9 methylation in the same promoter [106]. In addition to the regulation of BAT- or WAT-specific gene expression, LSD1 promotes BAT thermogenesis through repressing hydroxysteroid 11-β-dehydrogenase isozyme 1 (HSD11B1), a key glucocorticoid-activating enzyme, since glucocorticoid promotes lipid accumulation [108]. LSD1 binds to the promoter region of *Hsd11b1* independently of PRDM16 and demethylates H3K4me2. In a separate study, chemical inhibition of LSD1 was shown to block BAT differentiation accompanied by the repression of Wnt-β–catenin target genes [109]. Consistently, conditional deletion of *Lsd1* in newborn mice resulted in the inhibition of brown adipogenesis [109]. In another context, LSD1 is required for the formation and maintenance of thermogenic beige adipocytes that turn into white adipocytes with age, and PPARα mediates the effects of LSD1 on target genes [110]. Overall, it is clear that LSD1 is critical for adipogenesis and thermogenic gene regulation, although the exact mechanism for the thermogenic gene regulation remains to be elucidated further.

## Roles of LSD1 in neuronal physiology and neurodegenerative diseases

**Fig. 4:**
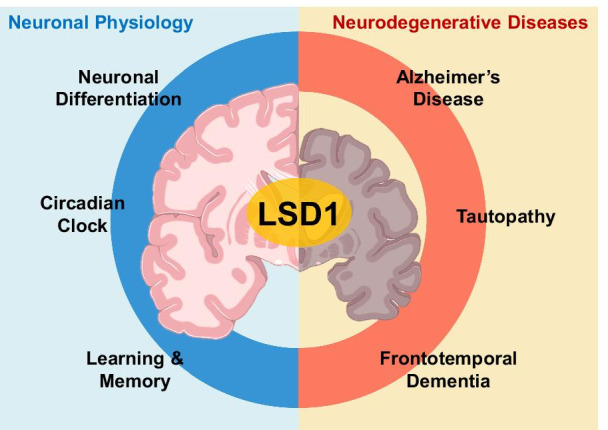
Roles of LSD1 in neuronal physiology and neurodegenerative diseases. LSD1 regulates gene expression in various neuronal physiology processes such as neuronal differentiation, circadian clock, learning, and memory. When LSD1 fails to control gene expression, neurodegenerative diseases such as Alzheimer’s disease (AD), frontotemporal dementia (FTD), and tauopathy occur

LSD1 is widely expressed in the brain and nervous system, regulating gene expression during various processes [111–115]. In particular, LSD1 functions as an epigenetic regulator of neural stem cell proliferation (Fig. 4). Its inhibitors pargyline and tranylcypromine lead to attenuated neural stem cell proliferation [112]. LSD1 was recruited to the promoter of TLX target genes for transcriptional repression, playing a novel role in regulation via TLX, an essential neural stem cell transcription factor [112]. LSD1 was targeted by E3 ubiquitin ligase for proteasomal degradation during neural differentiation in vitro as well as in vivo [111]. Further, LSD1 was described as critical for neuronal progenitor cell (NPC) maintenance during cortical development, controlling NPC differentiation by regulating H3K4 methylation of *ATN1* (encoding Atrophin 1, a protein related to dentatorubral-pallidoluysian atrophy) [113]. Pharmacological inhibition of LSD1 activity increased H3K4 methylation at the LSD1 binding site downstream of *ATN1*, leading to *ATN1* repression and NPC differentiation [113]. LSD1 was crucial for the neuronal differentiation of human fetal neural stem cells (hfNSCs) [114], being directly recruited to the *Hairy/enhancer-of-split related with YRPW motif-like* (*HEYL*) gene promoter and controlling H3K4me2 demethylation to repress HEYL expression during hfNSC neuronal differentiation [114]. Further, LSD1 co-repressor Rcor2 is expressed in the central nervous system and plays a key role in the epigenetic regulation of Sonic hedgehog (Shh) signaling during cortical development [115]. LSD1 and Rcor2 co-occupied the upstream regulatory regions of *Shh*, with the latter recruiting the LSD1 complex for the removal of H3K4me1 from these genes [115].

LSD1 is phosphorylated by PKCα in a circadian manner, and phosphorylated LSD1 interacts with CLOCK-BMAL1, a master transcriptional regulator, to facilitate transcriptional activation [52]. LSD1 phosphorylation-defective *Lsd1*^*SA/SA*^ mice exhibited attenuated expression of core clock genes, resulting in altered circadian rhythms and impaired phase resetting of the circadian clock [52]. Phosphorylated LSD1 was also reported to be involved in presynaptic plasticity as well as hippocampal learning and memory [116]. *Lsd1*^*SA/SA*^ mice exhibited significant upregulation of presynaptic function-related genes [116]. Further, LSD1 counteracted SETD1 activity during axonal branching and cortical synaptic dynamics, with *Setd1* depletion being accompanied by working memory deficits [117]. SETD1A, a lysine-methyltransferase, is a key schizophrenia susceptibility gene, and cognitive as well as circuitry deficits were observed in in *Setd1a*-deficient mice [117].

As LSD1 acts as a critical regulator of neuronal physiology, it has been reported that a lack of normal LSD1 function contributes to the occurrence of neurodegenerative diseases [118, 119]. LSD1 deletion in hippocampal and cerebral cortex neurons leads to paralysis, widespread hippocampal and cortical neurodegeneration, as well as learning and memory defects [118]. Ablation of LSD1 promoted expression changes in common neurodegeneration-associated genes in the degenerating hippocampus. LSD1 was specifically mislocalized to aggregates of pathological proteins such as pTau and pTDP-43 in Alzheimer’s disease (AD) and frontotemporal dementia (FTD) patients [118]. The role of LSD1 in neurodegeneration was also explored in mouse models. LSD1 sequestration and tau accumulation were observed in the nuclei of neurons in a tauopathy mouse model [118]. Further, LSD1 deletion in this mouse model affected tau-induced gene expression, while LSD1 overexpression rescued the neurodegenerative phenotype in the hippocampus of tauopathy mice [118]. Therefore, increasing the activity and expression of LSD1 may represent a promising therapeutic approach for the treatment of AD and FTD [119]. Although LSD1 has been reported as related to neurodegenerative disease, the detailed molecular mechanism of LSD1 remains to be elucidated. Therefore, the study of how LSD1 controls neurodegenerative disease-related gene expression through epigenetic regulation is a promising area for future studies.

## Concluding remarks

Since LSD1 is an epigenetic regulator involved in a variety of physiological processes, it has been associated with several diseases, including cancer as the most representative disease. LSD1 facilitates cancer cell survival and makes the microenvironment cancer-friendly; thus, inhibiting LSD1 function is an attractive strategy to suppress cancer. In vitro screening has identified many cancer cell lines that are not responsive to LSD1 inhibitors. However, LSD1 plays pleiotropic roles in tumor growth, invasion, metastasis, and drug resistance in vivo through the regulation of hypoxia, EMT, cancer stemness, and antitumor immunity. Therefore, the coverage of LSD1 inhibitors may be wider than that obtained from simple cell line screening. Accordingly, it seems necessary to develop a method that can test the efficacy of LSD1 inhibitors in a condition that best mimics the in vivo environment rather than a simple cell culture context. Combination therapy of an LSD1 inhibitor with other anti-cancer drugs is also an option worthy of consideration.

LSD1 is also involved in the development of various pathological conditions or diseases other than cancer. LSD1 maintains the balance of the inflammatory response by controlling the expression of various cytokine genes. In acute and severe inflammation conditions, LSD1 inhibition can restrict the spread of inflammation, as demonstrated in a CLP-mediated sepsis mouse model [15]. Thus, LSD1 inhibitors may be applied to alleviate certain types of inflammatory diseases. Moreover, LSD1 is important for the development, maintenance, and thermogenesis in brown and beige adipocytes, and loss of LSD1 function in adipocytes results in weight gain in mice. However, in this case, LSD1 inhibitors are not suitable for use, as LSD1 function is required to induce thermogenesis that is required to burn excess energy and thereby suppress obesity. A new method for increasing the expression or activity of LSD1 may be beneficial in this case. Moreover, LSD1 is widely expressed in the brain and nervous system, and plays a pivotal role in neuronal differentiation, the circadian clock, learning, and memory. As such, LSD1 plays a critical role in neuronal physiology, and when LSD1 dysfunction can lead to neurodegenerative diseases such as AD, FTD, and tauopathies. However, extensive studies are necessary to determine whether LSD1 is suitable as a drug target for brain and neurological disorders. Taken together, LSD1 acts as a key epigenetic regulator of gene expression that controls cellular homeostasis and can be a therapeutic target in certain diseases. Therefore, gaining an accurate understanding of the action mechanism of LSD1 is essential for overcoming several diseases.

## Data Availability

Not applicable.
